# Computed tomography and magnetic resonance imaging of multiple focal nodular hyperplasias of the liver with congenital absence of the portal vein in a Chinese girl: case report and review of the literature

**DOI:** 10.1186/s40001-014-0063-7

**Published:** 2014-11-26

**Authors:** Kun Zhang, Qingjun Wang, Haiyi Wang, Huiyi Ye, Aitao Guo, Weidong Duan

**Affiliations:** Department of Radiology, PLA General Hospital, #28 Fuxing Road, Beijing, 100853 China; Department of Pathology, PLA General Hospital, #28 Fuxing Road, Beijing, 100853 China; Department of Hepatobiliary Surgery, PLA General Hospital, #28 Fuxing Road, Beijing, 100853 China

**Keywords:** congenital absence of the portal vein, focal nodular hyperplasia, computed tomography, magnetic resonance imaging

## Abstract

**Background:**

Patients with congenital absence of the portal vein (CAPV) often suffer from additional medical complications such as hepatic tumors and cardiac malformations.

**Case presentation:**

Congenital absence of the portal vein (CAPV) is a rare malformation. We present a case of a 16-year-old Chinese girl with CAPV with multiple pathology-proven hepatic focal nodular hyperplasias (FNHs) and ventricular septal defect (VSD). The CT and MRI features of this case are described, and previously reported cases are reviewed.

**Conclusions:**

CAPV is a rare congenital anomaly and in such patients, clarifying the site of portosystemic shunts, liver disease, and other anomalies is critical for appropriate treatment selection and accurate prognosis determination. Close follow-up, including laboratory testing and radiologic imaging, is recommended for all CAPV patients.

## Background

In patients with congenital absence of the portal vein (CAPV), the mesenteric and splenic venous blood bypasses the liver through a congenital shunt vessel and drains into the systemic circulation, resulting in relatively poor perfusion of the liver [1]. To date, 78 such cases have been reported in the literature. Patients with CAPV often suffer from additional medical complications such as hepatic tumors and cardiac malformations. Here we report a case of CAPV with multiple hepatic focal nodular hyperplasias (FNH), which we studied with both computed tomography (CT) and magnetic resonance imaging (MRI).

## Case presentation

A 16-year-old Chinese girl was admitted to Chinese PLA General Hospital with left upper abdominal pain, which she had experienced for 1 month. Physical examination revealed no abnormalities. Laboratory investigations revealed moderate elevations of total bile (43.9 μmol/L; normal 0 to 10 μmol/L) and γ-glutamyl transpeptidase (89.5 U/L; normal 0 to 40 U/L), and mildly decreased serum albumin (33.2 g/L; normal 40 to 55 g/L). Serum aspartate aminotransferase (AST), alanine aminotransferase (ALT), total bilirubin, and alkaline phosphatase levels were normal. Serologic markers for hepatitis B and C virus were negative. A chest x-ray showed normal pulmonary vascularity. The patient did not have any neurological manifestations of hepatic encephalopathy, and her blood ammonia level was 56 μmol/L (normal 18 to72 μmol/L).

**Figure 1:**
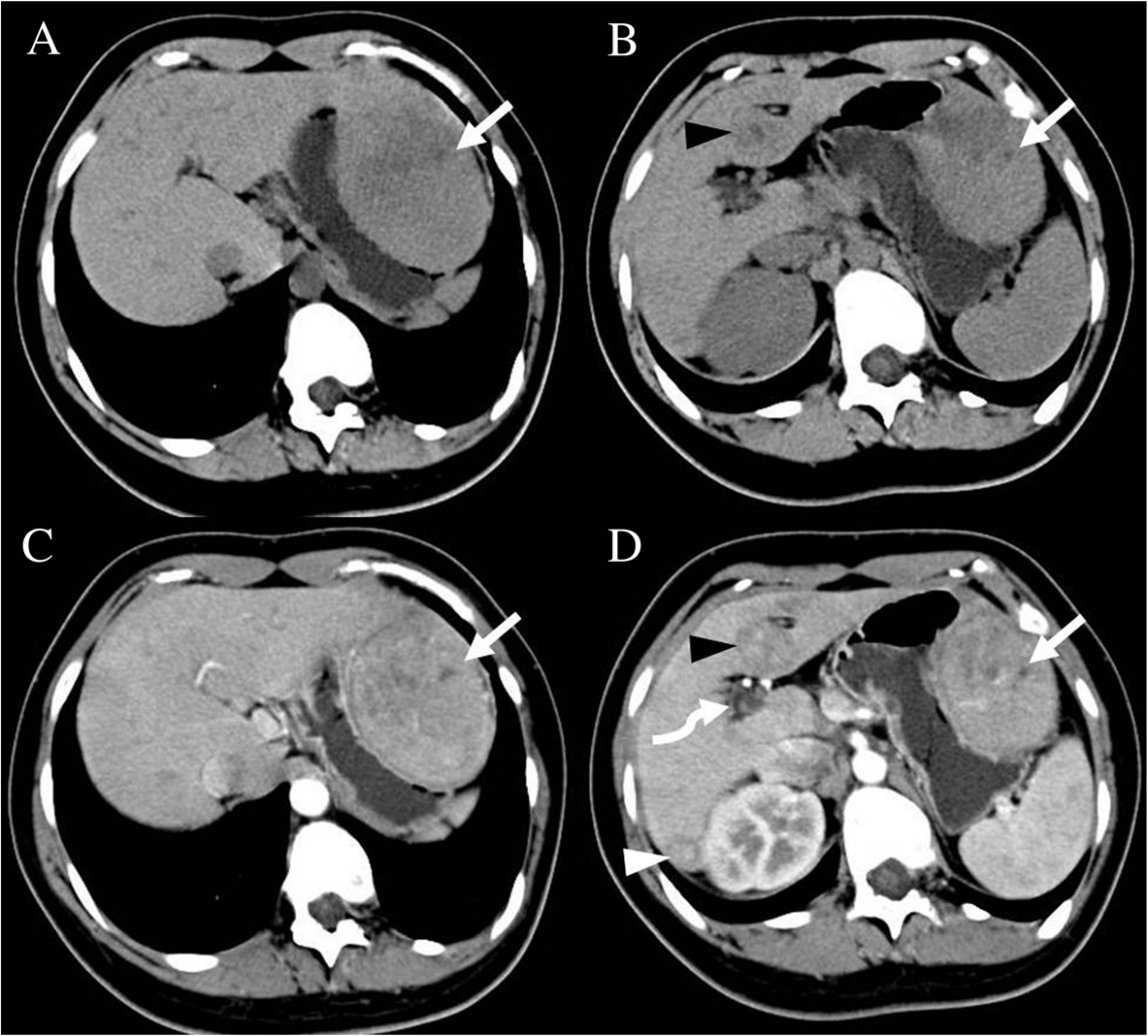
**Abdominal precontrast computed tomography (CT) show lesions in segment II and IV. A** and **B** show a large hypodense nodule (white arrow) in segment II and a small hypodense nodule (black arrowhead) in segment IV of the liver. The postcontrast CT images **(C and** **D)** show heterogeneously moderate enhancement of three lesions (white arrow, and black and white arrowheads) on arterial phase images. The intrahepatic portal vein was absent (white curved arrow).

**Figure 2:**
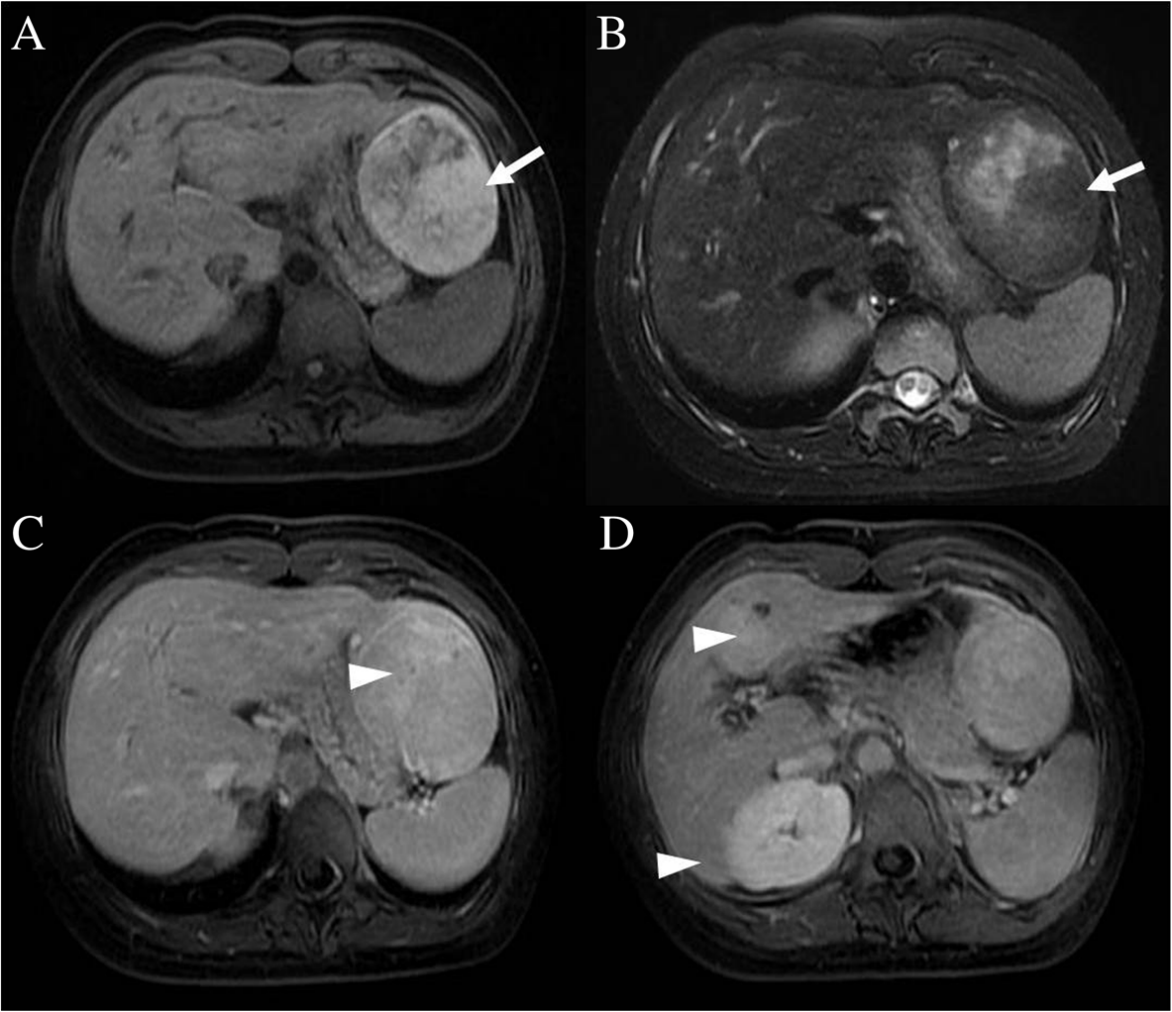
**Abdominal precontrast magnetic resonance imaging (MRI) show lesions in segment II.** Most of the tumor in segment II has heterogeneously high signal intensity on both fat suppressed T_2_-weighted images (**A**, white arrow) and T_1_-weighted images (**B**, white arrow). On delayed-phase images after gadopentetate dimeglumine administration, central delayed enhancement was noted in all three tumors (**C** and **D**, white arrowheads).

Precontrast abdominal CT revealed a large hypodense nodule in segment II (Figure 1A) and a small hypodense nodule in segment IV (Figure 1B) of the liver. Following intravenous administration of iodinated contrast, an additional lesion was seen in segment VI. Moderate enhancement was seen on arterial- and venous-phase images (Figure 1C,D). Precontrast MRI confirmed the presence of the three nodules in the liver. The largest one (segment II) measured 7.8 cm, and was markedly hyperintense on T_1_-weighted images (Figure 2A) and inhomogeneously hyperintense on T_2_-weighted images (Figure 2B). The other two lesions (segments IV and VI) measured 3.2 cm and 1.9 cm, respectively, and were heterogeneously hypointense on T_1_-weighted images and hyperintense on T_2_-weighted images. After intravenous injection of gadopentetate dimeglumine (Gd-DTPA, Magnevist; Schering AG, Berlin, Germany), all three lesions showed strong enhancement on arterial phase images. On portal-venous phase images, these lesions were moderately hyperintense compared with normal liver parenchyma due to washout of contrast agent, while on delayed-phase images, areas of delayed enhancement were seen in all three lesions (Figure 2C,D). Based on these CT and MRI findings, we diagnosed this case as multiple FNH.

**Figure 3:**
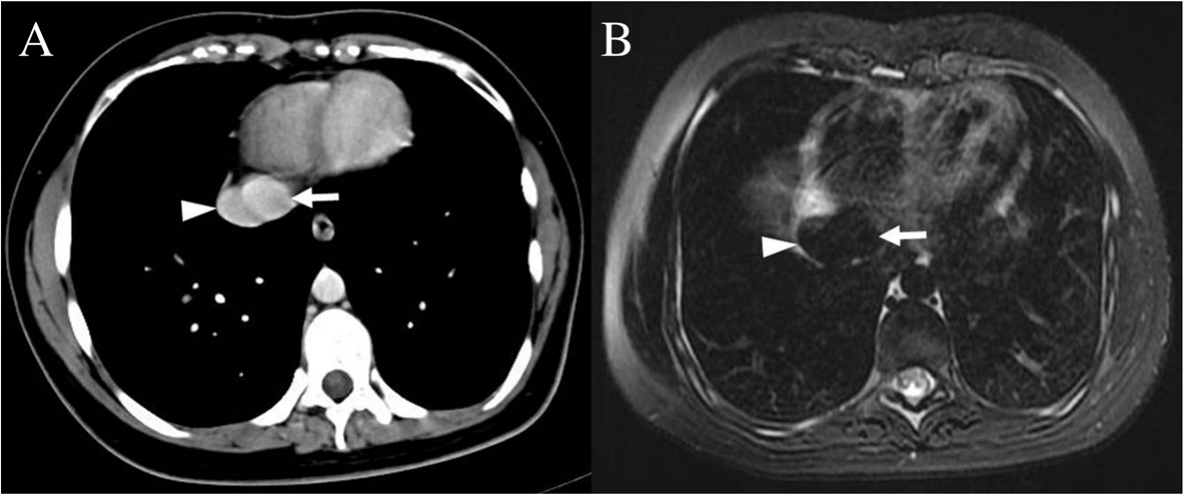
**Abdominal precontrast computed tomography (CT) and magnetic resonance imaging (MRI).** The portal trunk drains directly into the inferior vena cava (IVC) just below the level of the right atrium, shown on both postcontrast computed tomography (CT) **(A)** and T2-weighted magnetic resonance imaging (MRI) **(B)**. White arrowhead = IVC; white arrow = portal trunk.

During the radiologic workup of the patient, including both CT and MRI, no intrahepatic portal vein was seen. After joining of the middle and left hepatic veins, the portal trunk drained directly into the inferior vena cava (IVC) just below the level of the right atrium (Figure 3A,B). These vascular abnormalities were not identified prior to the hepatic surgery.

**Figure 4:**
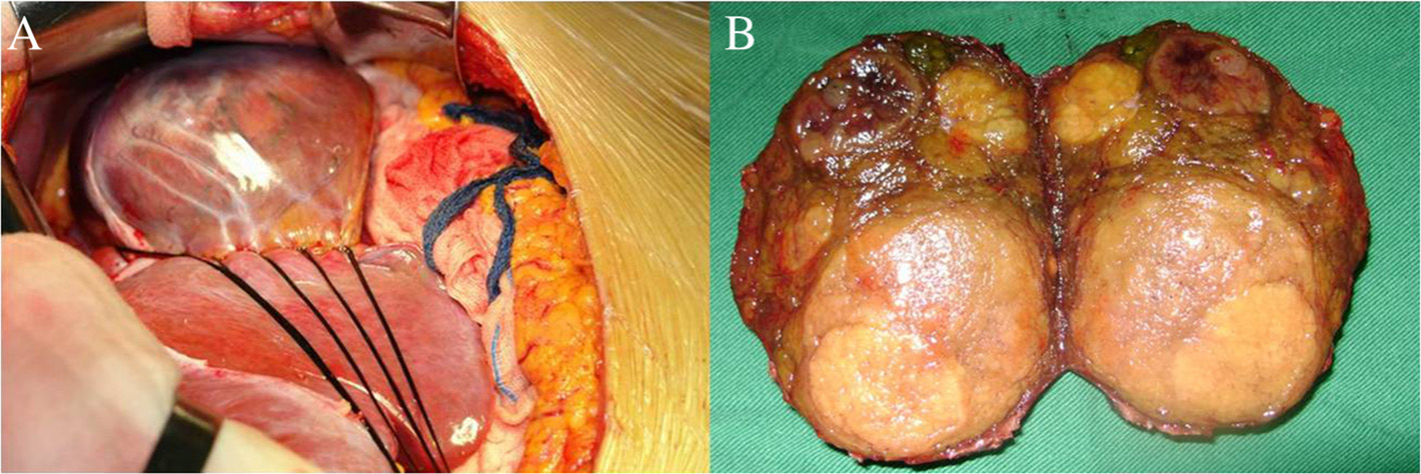
**Intraoperative exploration.** The largest tumor (segment II) is encapsulated by an intact capsule with rich subcapsular vessels **(A)**. The transverse section of the tumor in segment IV demonstrates multiple nodules in the capsule **(B)**.

**Figure 5:**
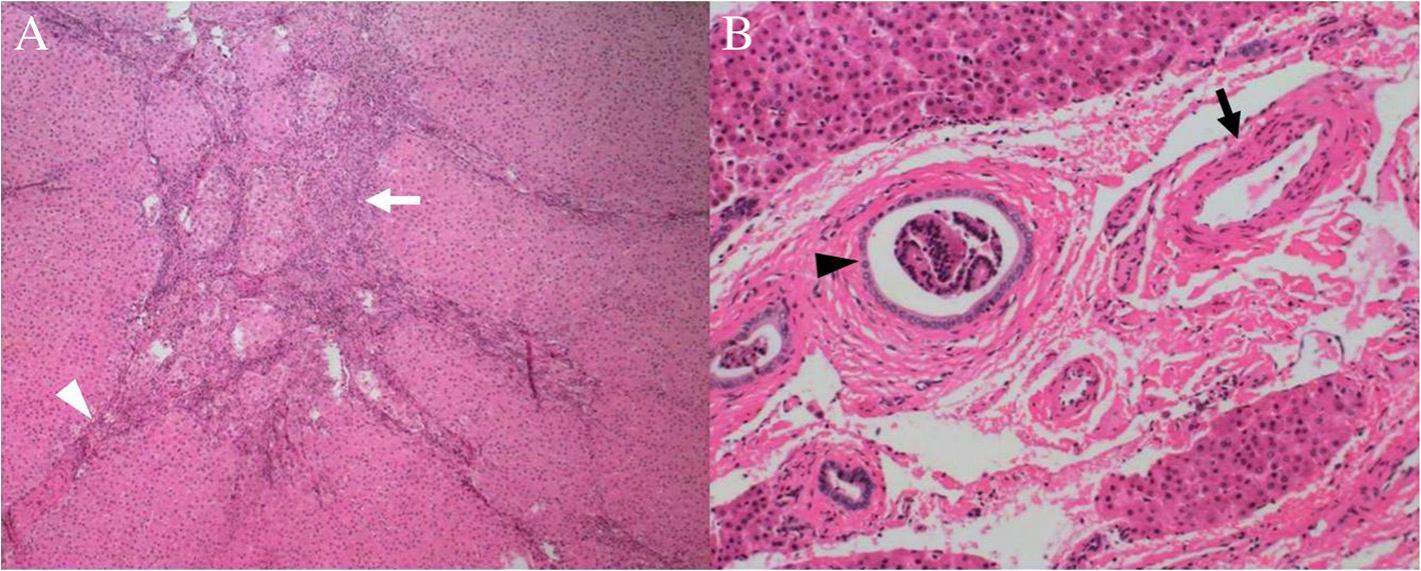
**Histological features of the classic focal nodular hyperplasia (FNH) in segment IV.** The tumor was subdivided into nodules by fibrous septa (**A**, white arrowhead) originating from a central scar (**A**, white arrow). The malformed arteries (**B**, black arrow) and bile ductular proliferation (**B**, black arrowhead) was detected. No vein was found. Hematoxylin & eosin, A × 40; B × 100.

**Figure 6:**
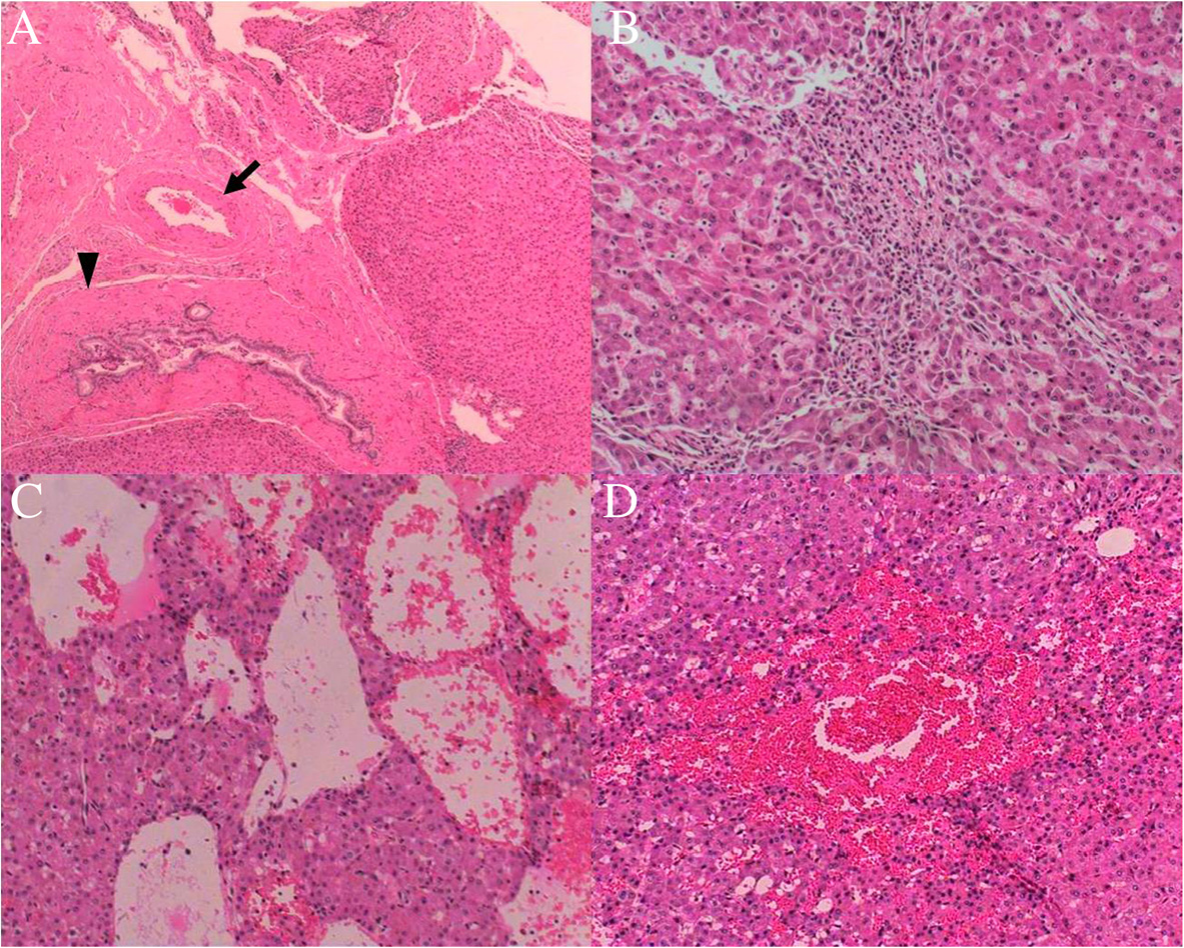
**Histological features of the telangiectatic focal nodular hyperplasia** **(FNH) in segment II.** The lesion contained fewer septa and no central scar. In a few areas, the FNH showed a short septum containing a small number of malformed arteries (**A**, black arrow) and some bile ductular proliferation (**A**, black arrowhead). Arteries were embedded in extracellular matrix with inflammatory cells **(B)**. Obvious sinusoidal dilatation **(C)** and peliosis **(D)** were observed. Hematoxylin and eosin, A 40X; B 100X; C 100X; D 100X.

Five days later, the patient underwent resection of the three hepatic tumors. During intraoperative examination, intrahepatic portal branches were not apparent. The largest lesion (segment II) protruded out of the liver and was encapsulated within an intact capsule with rich subcapsular vessels (Figure 4A). All three lesions were completely removed. The gross pathologic investigation from one lesion (segment IV) revealed multiple nodules in the capsule (Figure 4B). Microscopically, all three lesions comprised nodules of hepatocytes separated by fibrous septa containing numerous dystrophic arteries, proliferated bile ducts, and lymphocytic infiltration. The diagnosis of multiple FNH was confirmed; however, the histologic features of the two smaller FNH lesions (segments IV and VI) and the larger FNH lesion (segment II) differed. Aside from the lack of veins, the two smaller FNH lesions were characteristic of classic FNH: (a) abnormal nodular architecture, (b) radiating fibrous septa originating from a central scar, (c) malformed arteries, and (d) bile duct proliferation (Figure 5A,B). The larger FNH lesion (segment II) showed less central scar and fibrous septa and, more importantly, contained substantial sinusoidal dilatation, consistent with telangiectatic FNH (Figure 6).

Following surgery, liver ultrasonography (US) confirmed the CAPV and the presence of a portosystemic shunt, which fed directly into the IVC. Additionally, a ventricular septal defect (VSD) was observed using US.

**Figure 7:**
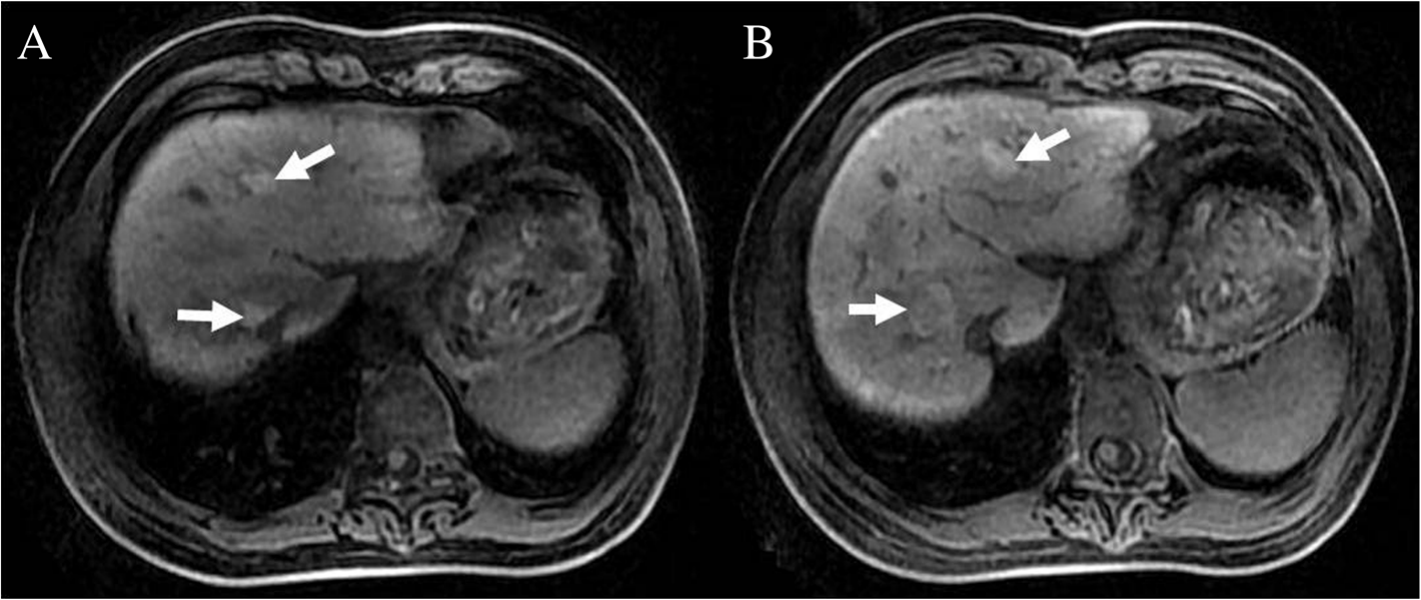
**Delayed hepatobiliary-phase of magnetic resonance imaging (MRI).** Following administration of gadobenate dimeglumine, additional hepatic tumors were found (**A** and **B**, white arrows).

Prior to discharge, the patient underwent an additional MRI with gadobenate dimeglumine (Gd-BOPTA, MultiHance; Bracco Imaging SpA, Milan, Italy), and additional hepatic nodules were found on delayed hepatobiliary-phase images obtained at one hour after contrast administration (Figure 7). These lesions were not seen on the CT examination or the MRI examination performed with gadopentetate dimeglumine.

## Discussion

Congenital absence of the portal vein is a rare malformation in which mesenteric and splenic venous flow bypasses the liver and drains into various sites in the systemic venous system via an extrahepatic portosystemic shunt. Girls are more commonly affected than boys, and the clinical presentation is diverse [2]. CAPV may be associated with other congenital cardiac, skeletal, or visceral anomalies [2]. The diagnosis of CAPV is readily made by US, CT, and MRI [2-4].

Embryologically, the portal vein arises from the vitelline venous system between the 4th and 10th week of gestation [1,5]. The intrahepatic portal vein originates from the superior link between the right and left vitelline veins; however, the extrahepatic portal vein arises from selective involution of the caudal part of the right and left vitelline veins [6]. Abnormal involution can lead to a preduodenal, prebiliary, or duplicated portal vein. Excessive involution can lead to complete or partial absence of the portal system [6]. As a result, mesenteric and splenic venous blood drains into renal veins, hepatic veins, or directly into the IVC, resulting in poor perfusion of the liver [2,7,8]. CAPV is often associated with hepatic tumors. The most common hepatic tumors reported were focal nodular hyperplasia (FNH). Other reported tumors included hepatoblastoma, hepatic adenoma, hepatocellular carcinoma and hemangioma.

To the best of our knowledge, a total 78 patients with CAPV have been described in the literature. The youngest was a fetus and the oldest was 64 years of age at the time of diagnosis [9,10]. The majority of CAPV patients were children (53/78, 67.9%) and female (53/78, 67.9%). The first case of portosystemic anomaly with congenital diversion of portal blood away from the liver was reported by Abernethy in 1793 [11]. Morgan *et al.* [12] defined complete portosystemic shunts not perfusing the liver via the portal vein as type I, and partial shunts with minimal portal perfusion to the liver as type II. Type I CAPV is further subclassified into types Ia and Ib based on the anatomy of the portal vein: in type Ia, the superior mesenteric vein (SMV) and splenic vein (SV) are not united to constitute a confluence; in type Ib, the SMV and SV join to form a common trunk. Whereas in type Ia the SMV and SV drain individually into the IVC, SMV, or renal vein [13-15], in type Ib the confluence trunk of the splenic and mesenteric vein usually drains into the suprarenal or suprahepatic IVC [3,16], or alternatively, it may flow into the hepatic vein, right atrium, iliac vein, inferior mesenteric vein, or renal vein [17,18].

In a review of the 78 cases of CAPV in the literature, we found that type Ib is more common than type Ia (65.4% versus 30.8%, respectively). In terms of gender, both types occur predominantly in females (type 1a: 73.8%; type 1b: 73.9%). The case described here was type Ib: the SMV and SV united to form a portal trunk, joining the middle and left hepatic veins, and draining into the IVC without passing through the liver.

Congenital absence of the portal vein with a complete loss of portal perfusion leads to developmental and functional alterations that predispose the liver to focal or diffuse hyperplastic or dysplastic changes. These conditions lead to an increased incidence of hepatic tumors or tumor-like conditions [1]. Of the 78 reported cases of CAPV, 34 had hepatic tumors. Hepatocellular carcinoma (HCC) was found in two cases [6,19], hepatoblastoma in three cases [14,20,21], adenoma in five cases [13,22-25], nodular regenerative hyperplasia (NRH) in five cases [16,26-28], and pathologically unconfirmed tumors in four cases [3,7,29,30]. However, focal nodular hyperplasia(FNH) was the most common hepatic tumor in patients with CAPV. Fifteen of the thirty-four reported cases with hepatic tumors involved FNH (Table 1), with four comprising solitary lesions [31-34], nine with multiple lesions [4,5,35-40], and one with an FNH lesion accompanied by additional tumors (HCC and adenoma) [41]. FNH and adenomas with a high risk of malignant transformation to HCC or hepatoblastoma [1]. Additionally, one reported case indicates a transformation from FNH to HCC in an adult patient with CAPV [42].

**Table 1:** **Summary of cases of congenital absence of the portal vein** (**CAPV) with focal nodular hyperplasia (FNH)**

**Case**	**Author**	**Year**	**Age (y)**	**Sex**	**Type**	**Hepatic Tumor**	**Other anomalies**	**Other disorders**
1	Morse [33]	1986	8	F	Ia	FNH (Solitary)	ASD, Goldenhar syndrome	LD
2	Matsuoka [34]	1992	22	F	Ib	FNH (Solitary)	Scoliosis with hemivertebra	LD
3	Motoori [39]	1997	18	F	Ib	FNH (Multiple)	Pancreatic tumor	LD
4	Guariso [32]	1998	8	F	Ib	FNH (Solitary)	Cystic kidney dysplasia	None
5	Wakamoto [31]	1999	6	F	Ib	FNH (Solitary)	PDA, Inguinal herniation	LD, Hyperammonemia
6	Kinjio [38]	2001	3	F	Ib	FNH (Multiple)	Congenital choledochal cyst	LD, Encephalopathy
7	Tanaka [37]	2003	16	F	Ib	FNH (Multiple)	Chronic active hepatitis	LD
8	De Gaetano [5]	2004	28	F	Ib	FNH (Multiple)	None	None
9	Schmidt [36]	2006	18	F	Ib	FNH (Multiple)	None	LD
10	Turkbey [4]	2006	10	F	Ia	FNH (Multiple)	None	LD
11	Morotti [41]	2007	8	F	Ia	FNH, HCC, adenoma	TS, Aortic coarctation	LD
12	Ringe [35]	2008	12	M	Ib	FNH (Multiple)	None	LD, Encephalopathy
13	Ringe [35]	2008	22	M	Ib	FNH (Multiple)	None	LD, Encephalopathy
14	Chandler [40]	2011	24	M	Ib	FNH (Multiple)	None	LD

ASD, atrioventricular septal defect; CAPV, congenital absence of the portal vein; FNH, focal nodular hyperplasia; HCC, hepatocellular carcinoma; LD, liver dysfunction; PDA, patent ductus arteriosus; TS, Turner syndrome.

Pathological investigation revealed one of the lesions to be telangiectatic FNH. The telangiectatic FNH lesion presented no central scar, fewer fibrous septa, and substantial sinusoidal dilatation. On T_1_-weighted images, the telangiectatic FNH showed obvious hyperintensity, which differed from the signal typically seen with classic FNH. These findings are consistent with a previous study by Attal *et al*. [43], who found that telangiectatic FNH lesions lack a central scar and tend to be hyperintense on T_1_-weighted images due to sinusoidal dilatation.

In a review of the fifteen cases of CAPV with hepatic FNH, MRI was used in nine cases. In five of these eight cases, lesions were hyperintense on T_1_-weighted images [4,5,31,37,38,40] and dilated sinusoids was seen histologically in 1 case. We suspect that some of these FNH lesions may have been telangiectatic and that the increased arterial hepatic flow by the hypertrophied hepatic artery may have played a role in their development.

Notably, delayed hepatobiliary phase MR imaging of the liver with gadobenate dimeglumine after surgery revealed numerous residual hepatic nodules not seen with gadopentetate dimeglumine. Given that delayed hepatobiliary phase MRI with gadobenate dimeglumine is known to improve the detection and characterization of FNH [44,45], we believe that these nodules most likely represented additional FNH lesions.

Because CAPV and cardiac malformations may result from a similar embryogenic insult, cardiac malformations are frequently observed in patients with CAPV, and may help compensate for the congestive effect of the portal vein in these patients. Skeletal abnormalities including scoliosis, hemivertebrae [26,34], and Goldenhar’s syndrome (oculo-auriculo-vertebral dysplasia) have also been described previously [14,33]. The majority of congenital cardiac abnormalities seen in CAPV patients are ventricular septal defects (VSD), atrial septal defects (ASD), open foramen ovale, and patent ductus arteriosus (PDA) [7,46-48]. The patient described here suffered from VSD, which was confirmed by US.

The majority of patients with CAPV have no signs or symptoms of portosystemic encephalopathy and only slightly abnormal liver function tests. Slight liver dysfunction was found in 41 patients, in spite of hyperammonemia being described in 11 patients and pulmonary hypertension (PA) in six patients. Our patient had slightly abnormal liver function, normal blood ammonia, and no encephalopathy. Wakamato *et al*. [31] suggested that the majority of patients with CAPV are not old enough to have portosystemic encephalopathy when described in the literature; nevertheless, the impact of portosystemic shunt on the central nervous system is serious, and its clinical potential should be considered in children with CAPV.

In general, US, CT, and MRI can all confirm the absence of the portal vein and visualize the portosystemic shunt. However, the vascular anomaly was not detected prior to surgery in this case. The use of US with echo-color Doppler may have been advantageous for depiction of the portal venous system and aberrant shunting. It is important to evaluate coexisting abnormalities, particularly hepatic tumors, with US, CT, and MRI. Although all three examination methods can be used as imaging tools in assessing coexisting hepatic tumors, delayed phase postcontrast MRI imaging with gadobenate dimeglumine may be the most sensitive for detection of small FNH lesions.

There are two treatment options available for patients with CAPV: close follow-up or liver transplantation. Close follow-up may be appropriate for patients with no signs or symptoms, or in those with only slight liver dysfunction. However, liver transplantation is indicated for patients with symptomatic CAPV refractory to medical treatment, especially those with associated hyperammonemia, portosystemic encephalopathy, hepatopulmonary syndrome (HPS), or hepatic malignant tumors [49-52].

## Conclusions

In conclusion, we describe a patient with CAPV, and typical and atypical (telangiectatic) FNH. This patient displayed slight liver dysfunction and cardiac anomaly. CAPV is a rare congenital anomaly and in such patients, clarifying the site of portosystemic shunts, liver disease, and other anomalies is critical for appropriate treatment selection and accurate prognosis determination. In addition, we describe the pathoradiologic features of FNH on the delayed phase of imaging with hepatobiliary-specific MRI contrast agents. Close follow-up, including laboratory testing and radiologic imaging, is recommended for all CAPV patients.

## Consent

Written informed consent was obtained from the patient for publication of this Case Report and any accompanying images. A copy of the written consent is available for review by the Editor-in-Chief of this journal.

## Abbreviations

CAPV: congenital absence of the portal vein
CT: computed tomography
FNHs: focal nodular hyperplasias
HCC: hepatocellular carcinoma
IVC: inferior vena cava
MRI: magnetic resonance imaging
NRH: nodular regenerative hyperplasia
SMV: superior mesenteric vein
SV: splenic vein
US: ultrasonography
VSD: ventricular septal defect
